# Microbiota-targeted interventions and clinical implications for maternal-offspring health: An umbrella review of systematic reviews and meta-analyses of randomised controlled trials

**DOI:** 10.7189/jogh.14.04177

**Published:** 2024-09-13

**Authors:** Bekalu Kassie Alemu, Ling Wu, Getnet Gedefaw Azeze, So Ling Lau, Yao Wang, Chi Chiu Wang

**Affiliations:** 1 Department of Obstetrics and Gynaecology, Faculty of Medicine, The Chinese University of Hong Kong, Hong Kong SAR; 2 Department of Midwifery, College of Medicine and Health Sciences, Debre Markos University, Ethiopia; 3 Department of Midwifery, College of Medicine and Health Sciences, Injibara University, Ethiopia; 4 LI Ka Shing Institute of Health Sciences; Faculty of Medicine, The Chinese University of Hong Kong, Hong Kong SAR

## Abstract

**Background:**

Microbes in the human body are the determinants of life-long health and disease. Microbiome acquisition starts in utero and matures during early childhood through breastfeeding. However, maternal gut dysbiosis affects the maternal-offspring microbiome interplay. Lines of evidence on dysbiosis-targeted interventions and their effect on maternal-offspring health and gut microbiome are inconsistent and inconclusive. Therefore, this study summarised studies to identify the most common microbiota-targeted intervention during pregnancy and lactation and to comprehensively evaluate its effects on maternal and offspring health.

**Methods:**

This umbrella review was conducted by systematically searching databases such as PubMed and the Web of Science from inception to 2 September 2023. The quality was assessed using the Assessment of Multiple Systematic Reviews-2 checklist. The Grading of Recommendations Assessment, Development, and Evaluation was used for grading the strength and certainty of the studies. The overlap of primary studies was quantified by the corrected covered area score.

**Results:**

A total of 17 systematic reviews and meta-analyses with 219 randomised controlled trials, 39 113 mothers, and 20 915 infants were included in this study. About 88% of studies had moderate and above certainty of evidence. Probiotics were the most common and effective interventions at reducing gestational diabetes risk (fasting blood glucose with the mean difference (MD) = −2.92, −0.05; *I^2^* = 45, 98.97), fasting serum insulin (MD = −2.3, −2.06; *I^2^* = 45, 77), glycated haemoglobin (Hb A1c) = −0.16; *I^2^* = 0.00)), Homeostatic Model Assessment of insulin resistance (HOMA-IR) (MD = −20.55, −0.16; *I^2^* = 0.00, 72.00), and lipid metabolism (MD = −5.47, 0.98; *I^2^* = 0.00, 90.65). It was also effective in preventing and treating mastitis (risk ratio (RR) = 0.49; *I^2^* = 2.00), relieving anxiety symptoms (MD = −0.99, 0.01; *I^2^* = 0.00, 70.00), depression in lactation (MD = −0.46, −0.22; *I^2^* = 0.00, 74.00) and reducing recto-vaginal bacterial colonisation (odds ratio (OR) = 0.62; *I^2^* = 4.80), and with no adverse events. It also effectively remodelled the infant gut microbiome (MD = 0.89; *I^2^* = 95.01) and prevented infant allergies. However, studies on pregnancy outcomes and preeclampsia incidences are limited.

**Conclusions:**

Our findings from high-quality studies identify that probiotics are the most common microbiome interventions during pregnancy and lactation. Probiotics have a strong impact on maternal and offspring health through maintaining gut microbiome homeostasis. However, further studies are needed on the effect of microbiota-targeted interventions on maternal cardiometabolic health, pregnancy, and neonatal outcomes.

**Registration:**

This umbrella review was registered with PROSPERO, CRD42023437098.

The human microbiome is a rapidly emerging field that reveals healthy aging through its interaction with body metabolism and immunity [1,2]. It plays a pivotal role in the developmental origin of health and disease and further benefits disease management, immunotherapy, and cancer control [3-6]. The occurrence and progression of the disease could be affected by the degree to which the microbiome is maldeveloped [7]. The human body microbiome is generated through intergenerational transfer and lifelong processes and is acquired predominantly from the maternal gut in early life. The maternal microbiome undergoes significant changes through advancing gestational age due to a wider range of physiological and hormonal adaptations [8-11].

Microbial transfer from the mother to the offspring starts in utero followed by exposure through the birth canal at birth and matures by breastfeeding [12,13]. An altered perinatal microbiome (dysbiosis) significantly affects the ability of infants to acquire a balanced early-life microbiome [14,15]. Gut dysbiosis is associated with gestational age at birth, delivery mode, and feeding pattern. It is also affected by maternal age, diet, body weight, medication, and environment [16-18]. In addition to its influence on offspring, dysbiosis is associated with maladaptation to pregnancy and may induce complications such as preeclampsia (due to its influence on the spiral artery remodelling) and gestational diabetes [19-22]. Microbiota-targeted interventions have been used for the management and prevention of gut dysbiosis and related health problems [23-25].

The commonly known microbiota-targeted interventions (MBTIs) include: 1) Probiotics **–** live nonpathogenic microorganisms that can increase the gastrointestinal tract microbial balance; these microorganisms are mostly of human origin and confer health benefits to the host and enable the prevention or improvement of some diseases when administered in adequate amounts [26,27]. It comprises *Lactobacillus* and *Bifidobacterium* species and Saccharomyces *boulardii* yeast and is regulated by dietary supplements and foods [27]. 2) Prebiotics – carbohydrates, that are nondigestible by human enzymes and are selectively metabolised by beneficial intestinal bacteria; they are designed to improve health by stimulating the numbers and/or activities of these bacteria [28,29]. 3) Synbiotics – mixtures of nonpathogenic microorganisms and substrate(s) selectively utilised by host microorganisms that confer health benefits to the host [30].

Thus far, MBTIs during pregnancy have been used in the management of metabolic disorders, including gestational diabetes (GDM), hypertension, and other pregnancy outcomes. There are several systematic reviews and meta-analyses of randomised controlled trials (RCTs) on MBTIs. However, the conclusions are inconsistent and contradictory to each other. For instance, the effects of MBTIs on GDM are debatable [31-34]. Similarly, the conclusions of MBTIs in maternal mental health management are contradictory [35,36]. Moreover, the type of intervention varied, and it may be difficult for clinical decision makers to first rate the most widely investigated MBTIs among pregnant women and determine their clinical significance for mother and offspring.

Based on these research gaps, we designed the following research questions. 1) Which MBTIs, and which microbiome categories have been investigated thus far during pregnancy and lactation? 2) Are these interventions clinically important for the mothers and babies, and to what clinical outcomes (if any)? 3) Can maternal supplementation of these interventions effectively orchestrate the infant gut microbiome and safe to the mother and baby? To answer these questions and provide evidence to clinical experts in maternal and foetal medicine, we conducted this umbrella review.

## METHODS

This umbrella review protocol was predesigned and registered in the international prospective register of systematic reviews, PROSPERO (CRD42023437098). The review was conducted and the report was presented according to the PRISMA (Preferred Reporting Items for Systematic Reviews and Meta-Analyses) flow structure [37] (Table S1 in the **Online Supplementary Document**).

### Search strategy and study selection

From lines of literature, microbiota-targeted interventions include probiotics, prebiotics, synbiotics, parabiotics, postbiotics, faecal microbiome transplants, and microbiome-containing diets such as probiotic yogurt [38-42]. However, for this study, we have prioritised and selected the most commonly available interventions in the literature named probiotics, prebiotics, and synbiotics.

**Figure 1:**
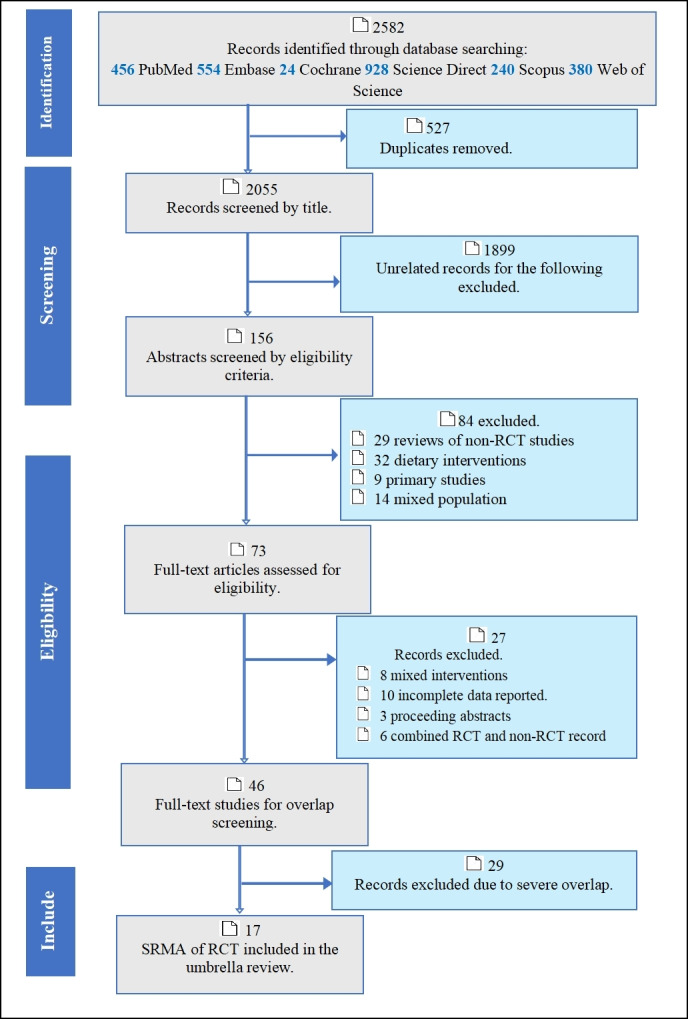
Selection of systematic reviews and meta-analyses using a PRISMA flowchart. RCT – randomised controlled trial, SRMA – systematic review and meta-analysis.

We systematically searched PubMed, the Web of Science, the Cochrane Library, EMBASE, Science Direct, and Scopus from inception to 2 September 2023. Since this study is the review of reviews, we did not search ClinicalTrials.gov for available studies. We designed the search terms based on the following contexts: 1) Population: Pregnant/lactating women ± infant pairs. 2) Intervention: probiotics, prebiotics, or synbiotics. 3) Control: placebo or no intervention. 4) Outcome: microbiome transfer to the infant's gut, maternal outcome, infant outcome, pregnancy outcome, and safety. Keywords and MeSH terms were combined with Boolean operators, and specific search engines were applied for each database (Table S2 in the **Online Supplementary Document**). The retrieved articles were exported to EndNote, and all titles and abstracts were screened to assess their eligibility for inclusion. Two authors (BKA and GGA) independently evaluated the full text of the articles for eligibility using the predetermined criteria and PRISMA flowchart (**Figure 1**). Disagreements between the two reviewers were discussed with the involvement of a third author (YW), and consensuses were reached. To account for studies missed in the original search, we manually scanned the reference lists of eligible articles.

Studies were considered eligible if they were systematic reviews or meta-analyses of randomised controlled trials that compared the effect of microbiota-targeted interventions on maternal and infant clinical outcomes, pregnancy outcomes, bacterial transfer to the baby and safety. PICOs (population: pregnant/lactating women ± infant pairs; intervention: probiotics, prebiotics or synbiotics; control: placebo or no intervention; and outcome: microbiome transfer to the infant's gut, safety to the mother and baby and maternal, infant and pregnancy outcomes) were taken as eligibility criteria to select the studies included in the umbrella review. All reviews published in the English language were included with no restriction on the year of publication. Narrative reviews, scoping reviews, reviews combining randomised controlled trials with observational studies such as cohort and case-control studies, and reviews of non-RCT studies were excluded.

Two authors (BKA and GGA) independently extracted the data using a pretested and standardised data extraction form, and discrepancies between the two authors were resolved by consulting a third author (YW). The data were extracted using an Excel form encompassing the first author’s name, year of publication, type of review, population, intervention, dosage, outcome, number of participants involved, risk assessment result, heterogeneity (*I^2^)*, effect model, publication bias (Egger’s/Begg’s test) and detailed results for each clinical outcome (**Table 1**). The clinical outcomes included gestational diabetes, group B* Streptococcus* colonisation, infant allergy, mastitis, maternal mental health, pregnancy outcome, safety during pregnancy and lactation, hypertension control during pregnancy, and bacterial transfer to the infant (**Table 1**).

**Table 1:** Characteristics of meta-analyses of RCTs included in this umbrella review

Outcome category	Authors	Interventions	Dosage CFU/d	Outcomes	Studies included	Sample size	Country	Conclusions
Pregnancy outcome	Othman et al., 2007 [43]	*L. rhamnosus GG, L. johnsanii; L. rhamnosus GR1 and L. reuteri RC-14; B. lactis Bb12*	10^7^	Preterm birth before 34 weeks	5	344	UK	Not conclusive for the impact on preterm birth.
Pregnancy outcome	Pérez et al., 2021 [44]	*L. reuteri; L.acidophilus; L. casei; L. salivarius; L. paracasei; L. casei L. fermentum; L. rhamnosus GG; L. delbrueckii bulgaricus; L. plantarum; B. breve; B. longum; B. infantis; L. sporogenes; B. animalis; B. bifidum; PF shermanii, S. thermophilus;*	1 × 10^8^ to 5 × 10^10^	Pregnancy outcomes (GA, BW, PB, C/S, macrosomia, SGA, LGA, miscarriage, and stillbirth	46	8363	Spain	Does not appear to influence perinatal outcomes.
GDM	Han et al., 2019 [32]	*L. rhamnosus GG, Lactobacilli and bifidobacteria; L salivarius; L. acidophilus; L. casei and L. rhamnosus HN001; S. thermophilus STY-31 and L. delbrueckii subsp Bulgaricus; breve, B. longum, B. infantis, B. lactis Bb12; B. bifidum;*	1 × 10^9^ to 1 × 10^10^	GDM and maternal metabolic changes during pregnancy	13	1139	China	It improved glucose and lipid metabolism
GDM	Mahdizade et al., 2022 [31]	*L. salivarius, L. paracasei,, L. acidophilus, L. plantarum, L.Paracasei, L delbrueckii subspBulgaricus; L. reuteri, L. fermentum; L. casei; L. Gasseri; L.rhamnosus GG, L. rhamnosus HN001, B. animalis subspLactis, B. bifidum; B.breve, B. longum, B. infantis, S. thermophilus,*	1 × 10^8^ to 4.5 × 10^11^	GDM	28	4806	Iran	Improved immune system function, glucose and lipid metabolisms, and reduced the risk of GDM
GDM	Chen et al., 2023 [34]	*Streptococcus, lactobacilli and Bifidum; Lactobacillus acidophilus, Lactobacillus rhamnosus HN001, B. longum, and B. bifidum; and B. animalis ssp;*	100 g/d probiotic yoghurt to 6 × 10^9^	Incidence of gestational diabetes	6	1861	China	Showed no benefits to prevent GDM
GDM	Masulli et al., 2020 [33]	*L. salivarius, L. paracasei, L. Acid, L. bulgaricus; L. Ramnosus; L. Casei; B. Lactis, B. Bifidum; B. BB12, S. Termop,*	1 × 10^9^ to 5 × 10^10^	Incidence of diabetes	17	3067	Italy	Do not reduce the incidence of GDM, but reduce FBS
Mental health	Desai et al., 2021 [35]	*L. rhamnosus GG, L. acidophilus; L. rhamnosus HN001; B. lactis BB; B. lactis*	6 × 10^9^ to 4.8 × 10^10^	maternal mental health (i.e. depression and anxiety and other mental health problems)	3	713	Ireland	Reduce anxiety symptoms
Mental health	Halemani et al., 2023 [36]	*L. rhamnosus; L. acidophilus, L. brevis, L. casei, L.salivarius, L. lactis; L. GG and L. plantarum, L. paracase, L. debrueckii and bulgaricus, and B. bifidum, B. lactis; B. infantis, B. breve, B. longum, B. longum; B. animalis; L. reuteri DSM, B. breveM-16v (1-104 CFU / 100 ml IG2 = 0.8 g / 100 ml, scGOS / lcFOS and B. breve M-16v (1-106 CFU / 100 ml). B. breve PB04 and bifidum, scGOS/Lcfos (synbiotic) or scGOS / lCFOS (prebiotic); bovin milk derived oligosaccharides; ScGOS/lcFOS and S. thermophilus; P.freudenreidii ssp, shermanii JS;*	5 × 10^6^ to 9 × 10^11^	Anxiety and depressions of pregnant and lactating women	14	3307	India	Reduced anxiety and depression symptoms
GBS colonization	Menichini D, 2022 [45]	*L. rhamnosus GR-1 and L.reuteri RC-14; L. jensenii Lbv116; L. crispatus Lbv88; L. rhamnosus Lbv96; L. gasseri Lbv150*	1 × 10^8^ to 5.4 × 10^9^	GBS colonization	5	583	Italy	Effectively prevent and manage GBS rectovaginal colonization
Infant allergies	Kuang L Y, 2020 [46]	*L. GG; L. rhamnosus; L. reuteri ATCC; and L. acidophilus, and L. lactis; L. salivarius, L. paracasei, B. bifidum, B. lactis, B. animalis subsp lactis, B. bifidum; B. breve Bbi99, B. infantis, B. bifidum, B. Longum, PF ssp. Shermanii*	1 × 10^8^ to 5 × 10^10^	atopic eczema, eczema, allergic disease, IgE-associated allergic disease, asthma and sensitisation	18	4356	China	Effective for atopic eczema, eczema, gestational age, death and NEC
Infant allergies	Colquitt et al., 2022 [47]	*L. rhamnosus GG; and L. acidophilus La-5; L. rhamonosus LPR, L. rhamnosus HN001 L. paracasei; B. animalis BB12, B. longum*	NR	atopic disease	6	3263	UK	Reduce the risk of infant AD or eczema.
Lactational mastitis	Yu et al., 2022 [48]	*L. salivarius CECT5713, L. gasseri CECT5714; L. fermentum CECT 5716; L salivarius PS2,*	1 × 10^9^ to 1 × 10^10^	incidence of lactating mastitis and breast pain, bacterial count in milk.	6	1197	China	To certain extent reduce the incidence and symptoms of mastitis
Safety	Dugoua et al., 2009 [49]	*L. reuteri; LGG; L. johnsonii, Probiotics & galactooligosaccharides;*	1 × 10^8^ to 2 × 10^10^	Safety; C/S rate, BW, & GA.	8	1546	Toronto	No malformations and other adverse events were reported.
HTN in pregnancy	Movaghar R, 2022 [50]	*L.acidophilus, L. plantarum, L. fermentum L. gasseri; L. delbrueckii bulgaricus LBY; L. casei, L. salivarius; B. bifidum; B.BB-12, S. thermophilus, FOS*	38.5 mg to 1 × 10^10^	Systolic blood pressure, Diastolic blood pressure, Preeclampsia	5	428	Iran	No significant difference in preeclampsia
BT	Moore et al., 2020 [51]	*Lacidophilus La-5, L. rhamnosus GG; Lactococcus lactis (Lc Lactis); B. animalis subsp lactis Bb-12; B. bifidum W23; B. lactis;*	1 × 10^9^ to 5 × 10^10^	Bacteria transfer to the infant	3	278	Ireland	Inconclusive evidence of vertical transfer of bacteria
BT	Martin et al., 2022 [52]	*L. acidophilus, L. plantarum, L. paracasei, L.delbrueckii subsp Bulgaricus, B. longum, B. breve, B. lactis; B. infantis, and S. thermophilus*	1 × 10^4^ to 9 × 10^11^	Bacteria transfer to the infant	12	2285	Spain	Had beneficial effects on the gut microbiota
BT	Bekalu et al., 2023 [53]	*L.salivarius and L.gasseri; L reuteri; L. rhamnosus GG; L.fermentum; L.rhamnosus GR-1; L.acidophillus; B. animalis; S.boulardii; LPR + B. longum; B. lactis; B.Actiregularis; S. thermophilus, saccharomyces boulardii*	NR	Bacteria transfer	24	2761	Hong Kong	Effectively modulate infant gut microbiome

#### Quality assessment and grading of certainty of evidence

The methodological quality of the included reviews was evaluated using a measurement tool to assess systematic reviews (AMSTAR-2: Assessment of Multiple Systematic Reviews checklist) [54]. Using the GRADE- Grading of Recommendations Assessment, Development, and Evaluation system, we evaluated the strength and certainty of the evidence in the included reviews. Since our umbrella review included only RCTs, the grading started from ‘High’ certainty and was subsequently assessed in five stages named ‘risk of bias’, ‘inconsistency/heterogeneity’, ‘indirectness’, ‘imprecision’ and ‘publication bias’ [55]. The certainty of evidence of each review was downgraded one stage when it had ‘serious’ inconsistency/indirectness/imprecision or ‘likely’ for publication. When judged as ‘very serious’ or ‘very likely’ to these domains, two stages were demoted at a time (Table S3 in the **Online Supplementary Document**). Two reviewers (BKA and GGA) evaluated the methodological quality and strength of each study included.

#### Overlap management

After systematic screening of the studies by title, abstract and full article, primary outcome-based thematisation of each record was performed according to the following categories: effect of microbiota-targeted interventions during pregnancy and lactation on 1) gestational diabetes, 2) Group B *Streptococcus* colonisation (GBS), 3) infant allergies (dermatitis), 4) lactational mastitis, 5) maternal mental health, 6) pregnancy outcome, 7) safety during pregnancy, 8) bacterial transfer to the baby, and 9) hypertension control during pregnancy. After thematisation, a citation matrix (graphical cross-tabulation) of the overlapping systematic reviews (in columns) and the included primary studies (in rows) was generated for reviews found to have overlapping associations (Tables S4–12 in the **Online Supplementary Document**) [56]. A citation matrix allows the degree of overlap to be quantified with a measure known as the corrected covered area (CCA), which was calculated as (N−r)/(rc − r), where N represents the number of publications included in evidence synthesis (or the number of ticked boxes in the citation matrix), r represents the number of rows, and c denotes the number of columns. Overlap was categorised as very high (CCA>15%), high (CCA 11–15%), moderate (CCA 6–10%), or slight (CCA 0–5%). The corrected covered area is a promising method for quantifying the degree of overlap between two or more reviews and helps the decision process on how to address overlap when it occurs [57,58]. The highest degree of overlap was found for the gestational diabetes theme in which a relatively greater number of studies fulfilled the eligibility criteria (Table S3, Figure S1 in the **Online Supplementary Document**). When a high degree of overlap (CCA≥11%) between two or more reviews was found, the most recent study that had the highest number of studies or participants and assessed with the AMSTAR-2 quality assessment tool as a better quality was prioritised for inclusion in the overview. In the case of slight or moderate overlap (CCA≤10%), both reviews were included in the analysis.

Data from the systematic reviews and meta-analyses that met the inclusion criteria were synthesised via a narrative synthesis, and findings from reviews that reported a meta-analysis were presented in tabular presentations and forest plots. We also presented summary tables describing the review characteristics. Alluvial graphs were constructed to summarise clinical outcomes in mothers and infants, interventions (probiotic species, prebiotics, and synbiotics), genus categories of bacterial species, and types of microbiota-targeted interventions. Mean differences for continuous outcome measures, and RRs or ORs for binary outcome measures were used to measure the impact of microbiota-targeted interventions on different outcomes.

## RESULTS

The systematic search resulted in a total of 2582 records were retrieved and exported to EndNote software for screening. After 527 duplicates were removed, thorough screening was performed to yield 73 articles for full-length review. After excluding reviews for reasons such as mixed intervention, mixed designs, and severe overlap, a total of 17 systematic reviews and meta-analyses were included in the final umbrella review (**Figure 1**). Of these, three (n = 3) were only systematic reviews, and the remaining 14 were systematic reviews and meta-analyses of randomised controlled trials. Within the 17 included review articles that had a tolerable range of overlap (none to moderate), 39 113 women and 20 915 infants in 219 RCTs were involved.

According to the critical quality assessment criteria, all the included reviews except the one [53] did not report the full list and reasons of excluded studies, and considered as low quality. Additionally, owing to noncritical criteria, the funding sources of the included primary studies (RCTs) were not mentioned in any of the reviews in this umbrella review. In other domains, all reviews were evaluated as qualified. According to the GRADE system, approximately 64.7% (n = 11) of studies had moderate certainty of evidence, 23.5% (n = 4) involved high certainty of evidence, and 11.8% (n = 2) involved low certainty of evidence (**Table 2**, Table S4 in the **Online Supplementary Document**). The overall quality of included SRMAs was considered to be the standard, and the conclusion can be accepted [59].

**Table 2:** Characteristics of reviews included in the umbrella review

Review	Review type	Population	Outcome	No. of studies in the review	No. of studies in the MA	No. of studies with low RoB	Risk of bias	Certainty
Othman et al., 2007 [43]	Cochrane SRMA	Pregnant	Preterm birth	5	5	NR	High risk	Moderate
Dugoua et al., 2009 [49]	SRMA	Pregnant	Safety	8	8	NR	Low risk	Moderate
Han et al., 2019 [32]	SRMA	Pregnant	GDM	13	13	13	Low risk	High
Masulli et al., 2020 [33]	SRMA	Pregnant	GDM	17	17	17	Low risk	Moderate
Kuang L Y, 2020 [46]	SRMA	Pregnant	Infant allergies	18	18	NR	Unclear	High
Moore et al., 2020 [51]	SR	Pregnant	Bacterial transfer	3	0	3	Low risk	High
Desai et al., 2021[35]	SRMA	Pregnant	Mental health	3	3	3	Low risk	Moderate
Pérez et al., 2021[44]	SRMA	Pregnant	Pregnancy outcome	46	25	18/46	High risk	Moderate
Mahdizade et al., 2022 [31]	SRMA	Pregnant	GDM	28	28	28	Low risk	Moderate
Chen et al., 2023 [34]	SRMA	Pregnant	GDM	6	6	NR	Unclear	Low
Menichini D., 2022 [45]	SRMA	Pregnant	GBS colonization	5	5	NR	Unclear	Moderate
Colquitt et al., 2022 [47]	SR	P & L	Any atopic disease	6	0	5	Low risk	Low
Yu et al., 2022 [48]	SRMA	Lactating	Mastitis	6	6	NR	Unclear	Moderate
Movaghar R, 2022 [50]	SRMA	Pregnant	Preeclampsia	5	5	5	Low risk	Moderate
Martin et al., 2022 [52]	SR	P & L	Bacterial transfer	12	0	7	Low risk	High
Halemani et al., 2023 [36]	SRMA	P & L	Mental health	14	4	14	Low risk	Moderate
Bekalu et al., 2023 [53]	SRMA	P & L	Bacterial transfer	24	21	21	Low risk	Moderate

GDM – gestational diabetes mellitus, I/C – intervention/control, MA – meta-analysis, NR – not reported, P&L – pregnant and lactating women, RoB – risk of bias, SR – systematic review, SRMA – systematic review and meta-analysis

**Figure 2:**
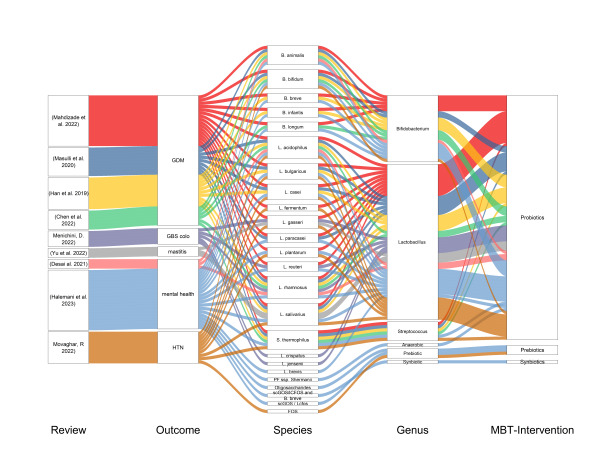
Summary of microbiota-targeted interventions on maternal clinical outcomes. GDM: gestational diabetes mellitus, GBS colo – Group B* Streptococcus* colonisation, HTN – hypertension during pregnancy.

Microbiota-targeted interventions (MBTIs) were explained in terms of bacterial strains, species type, genus category, intervention category, and composition profile (particularly for prebiotics and synbiotics). For ease of understanding and presentation, we classified interventions into two parts based on the clinical outcomes reported. The first category included interventions administered to evaluate maternal clinical outcomes (gestational diabetes, group B* Streptococcus* colonisation, mastitis, maternal mental health, and hypertension). To evaluate these outcomes, 20 bacterial species (*B. animalis, B. bifidum, B. breve, B. infantis, B. longum, L. acidophilus, L. brevis, L. bulgaricus, L. casei, L. crispatus, L. fermentum, L. gasseri, L. jensenii, L. paracasei, L. plantarum, L. reuteri, L. rhamnosus, L. salivarius, PF ssp. Shermanii, and S.thermophilus*), three prebiotic ingredients (fructo-oligosaccharides, oligosaccharides, short-chain galactooligosacCharides/long-chain fructooligosaccharides) and one prebiotic-probiotic combination (synbiotic) intervention (scGOS/lcFOS and *B. breve*) were administered during pregnancy. The other main outcome that could not be classified under maternal or baby clinical outcome was safety during pregnancy and interventions for the safety evaluation comprised (L. reuteri, LGG, L. johnsonii and galactooligosaccharides). The bacterial species used in the maternal clinical outcome evaluation were under the genera *Lactobacillus*, *Bifidobacterium*, *Streptococcus*, and *Anaerobes* (*PF ssp. Shermanii*), and all of these were again categorised as probiotics (**Figure 2**).

**Figure 3:**
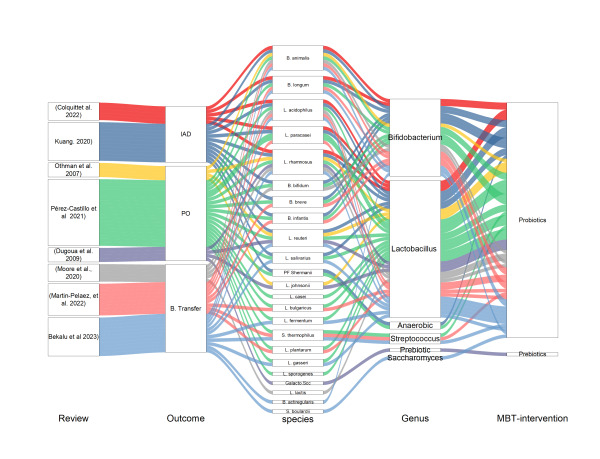
Summary of microbiota-targeted interventions on infant clinical outcomes. B.Transfer – bacterial transfer from mother to infant, IAD – infant allergic disease, PO – pregnancy outcome.

Interventions targeting to influence babies’ clinical outcomes were 22 probiotic bacteria under genera *Bifidobacterium* (*B. actiregularis, B. animalis, B. bifidum, B. breve, B. infantis, B. longum) Lactobacilus (L. acidophilus, L. bulgaricus, L. casei, L. fermentum, L. gasseri, L. johnsanii, L. lactis, L. paracasei, L. plantarum, L. reuteri, L. rhamnosus, L. salivarius, L. sporogenes), Anaerobes (PF Shermanii), saccharomyces (S. boulardii),* and *Streptococcus (S. thermophilus), and one prebiotic ingredient (*Galacto.Scc) (**Figure 3**).

In the reviews and meta-analyses included, probiotics, prebiotics and synbiotics were used as microbiota-targeted interventions under the nine main clinical outcomes. These clinical outcomes can be categorised as maternal, babies’ and pregnancy outcomes.

### Effect of MBTIs on maternal outcomes

**Figure 4:**
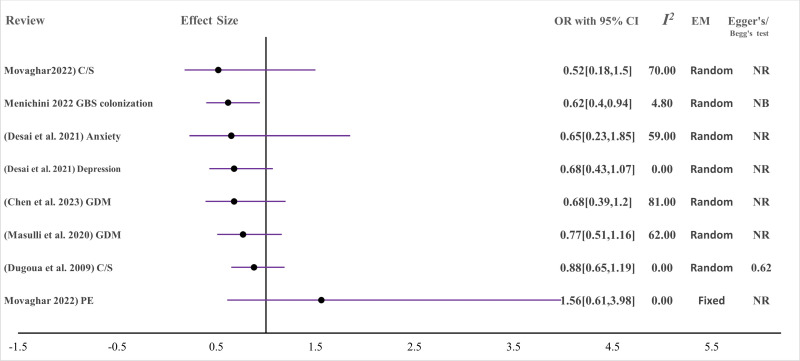
Effect of MBTIs on pregnancy and maternal clinical outcomes with binary outcomes (measured with OR (odds ratio)) (*I^2^*: heterogeneity (*I*-squared). C/S – Caesarean section, EM – effect model, GBS – Group B *Streptococcus*, GDM – gestational diabetes mellitus, *I^2^* –* I* squared, MBTI – microbiota-targeted interventions NB – no bias from funnel plots, NR – not reported, PE – preeclampsia.

**Figure 5:**
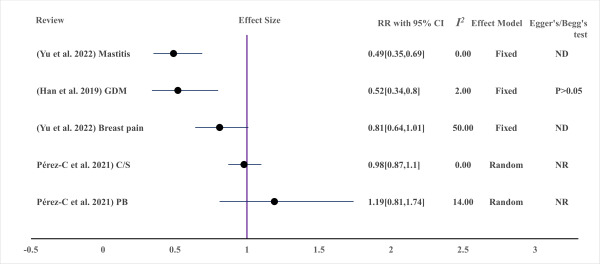
Effect of MBTIs on maternal clinical outcomes with binary outcomes (measured in RR (risk ratio)). GDM – gestational diabetes mellitus, C/S – Caesarean section, MBTI – microbiota-targeted interventions, ND – not done due to small studies, NR – not reported, PB – preterm birth.

A. Gestational diabetes mellitus (GDM) (n = 4) [31-34]: to summarise the effect of microbiota-targeted interventions (MBTIs) during pregnancy on the prevention and management of gestational diabetes, four systematic reviews and meta-analyses (three out of four studies with moderate to high certainty and strength of evidence) randomised controlled trials (n = 64) were included. About 64 RCTs with moderate overlap (CCA = 10.8%) and with 10 903 participants were included. To summarise the effect of MBTIs on gestational diabetes incidence, key variables such as glycaemic status, lipid profile, and inflammatory and oxidative stress marker levels were taken as outcome measures. All the included studies that assessed the impact of MBTIs on gestational diabetes (fasting blood glucose) as their primary outcomes pooled the mean fasting blood glucose (FBS) level difference and two (n = 3) [31,32,34] of them demonstrated a significant impact in controlling FBS. Another study (n = 1) [33] showed a reduction effect but not significant. Three of them evaluated the effect of interventions on fasting serum insulin (FSI) levels and demonstrated a significant reduction among the intervention groups. The intervention was also effective at controlling homeostasis model assessment insulin resistance (HOMA-IR). Regarding the lipid profile, microbiota-targeted interventions were effective at reducing very low-density lipoprotein (VLDL), total cholesterol levels, and triglyceride levels. The inflammatory markers tumour necrosis-alpha (TNF-α) and interleukin-6 (IL6) were significantly lower in the intervention group than in the control group. However, there was no difference between the two groups on the hs-CRP levels. The mean differences in nitric oxide bioavailability, total antioxidant capacity and cellular glutathione concentrations were significantly greater among the intervention group. In contrast, the mean serum concentration of malondialdehyde decreased more in the intervention group than in the control group (**Table 3**). Although the difference was not significant, the odds of occurrence of GDM among participants in the intervention group was lower than that among participants in the control group (**Figure 4**) [31,33] Similarly, the risk of diabetes was lower in the intervention group (**Figure 5**) [32].

**Table 3:** Effect of microbiota-targeted interventions on gestational diabetes markers (effect size with confidence intervals)

Outcome category	Author Year	Outcome measured	ES (Means)	95%CI	*I^2^*	Effect model	Egger's test
	Mahdizade et al., 2022	FBS	–2.92	–5.33, –0.51	98.97	Random	0.0042
	Masulli et al., 2020	FBS	–1.05	–1.95, 0.16	45	Random	NR
	Han et al., 2019	FBS	–0.11	–0.16, –0.05	71	Random	>0.05
	Chen et al., 2023	FBS	–0.05	–0.29, –0.19	75	Random	NR
	Mahdizade et al., 2022	FSI	–2.30	–4.10, –0.50	55.89	Random	0.3496
	Masulli et al., 2020	FSI	–1.63	–2.56, –0.71	45	Random	NR
	Han et al., 2019	FSI	–2.06	–2.98, –1.15	77	Random	>0.05
	Mahdizade et al., 2022	Hb A1c	–0.16	–0.39, 0.07	0	Random	NR
	Mahdizade et al., 2022	HOMA-IR	–0.59	–0.98, –0.19	47.8	Random	0.1597
	Mahdizade et al., 2022	HOMA-IR	–20.55	–35.50, –5.63	0	Random	NR
	Masulli et al., 2020	HOMA-IR	–0.19	–0.44, 0.05	72	Random	NR
	Han et al., 2019	HOMA-IR	–0.38	–0.54, –0.21	64	Random	>0.05
	Mahdizade et al., 2022	Nitric Oxide	1.30	–0.56, 3.25	0	Random	0.9365
	Chen et al., 2023	OGTT	–0.07	–0.27, 0.13	62	Random	NR
	Mahdizade et al., 2022	QUICKI	0.01	0.0, 0.02	0	Random	0.6804
	Mahdizade et al., 2022	C-Peptide	0.08	–0.24, 0.4	0	Random	NR
Inflammatory and oxidative stress marker	Mahdizade et al., 2022	TNF-α	–1.07	–1.72, –0.42	NR	Random	NR
	Mahdizade et al., 2022	Interleukin-6 (IL6)	–0.77	–1.2, –0.34	NR	Random	NR
	Mahdizade et al., 2022	MDA	–0.48	–0.77, –0.2	0	Random	0.8535
	Mahdizade et al., 2022	hs-CRP levels	–252.36	–780.82, 276.1	100	Random	0.0001
	Mahdizade et al., 2022	GSH/glutathione	30.14	6.59, 66.88	0	Random	0.5055
	Mahdizade et al., 2022	TAC	70.76	20.8, 120.72	0	Random	0.9033
Lipid metabolism	Mahdizade et al., 2022	VLDL levels	–5.47	–10.07, –0.86	0	Random	0.6576
	Mahdizade et al., 2022	Cholestrol	–0.32	–0.65, 0.01	2.25	Random	0.6211
	Han et al., 2019	HDL	–0.13	–0.34, 0.07	33	Random	>0.05
	Mahdizade et al., 2022	HDL	0.13	–0.14, 0.41	56.4	Random	0.0942
	Mahdizade et al., 2022	LDL	0.98	–1.52, 3.48	95.31	Random	0.9903
	Han et al., 2019	LDL-cholesterol	–0.45	–0.97, 0.06	89	Random	>0.05
	Han et al., 2019	Total cholesterol	–0.56	–1.07, –0.05	89	Random	>0.05
	Mahdizade et al., 2022	Triglycerides	0.04	–1.45, 1.53	90.65	Random	0.0186
	Han et al., 2019	Triglycerides	–0.66	–1.28, –0.04	92	Random	>0.05

FBS – fasting blood glucose, FSI – fasting serum insulin, GSH – glutathione, Hb A1C – haemoglobin A1c, HDL – high-density lipoprotein, 2h OGTT – two hours oral glucose tolerance test, HOMA-B – homeostasis model assessment of β-cell function, HOMA-IR – homeostasis model assessment of insulin resistance, hs-CRP – high-sensitivity C-reactive protein, LDL – low-density lipoproteins, MDA – malondialdehyde, VLDL – very low-density lipoprotein, NR – not reported, QUICKI – quantitative insulin-sensitivity check index, TAC – total antioxidant capacity, TNF-α – tumour necrosis factor alpha

B. Group B *Streptococcus* (GBS) colonisation: to measure this outcome, due to the very high overlap between available reviews, only one recent review with a comparably larger number of primary studies (n = 5) and moderate certainty of evidence was included [45]. In this review, a total of 583 pregnant women were evaluated for GBS colonisation after MBTI supplementation during pregnancy (**Figure 4**), and the intervention was associated with decreased GBS rectovaginal colonisation and a safe perinatal profile, which can be explained by no adverse events reported in each study in both the mother and the baby.

**Figure 6:**
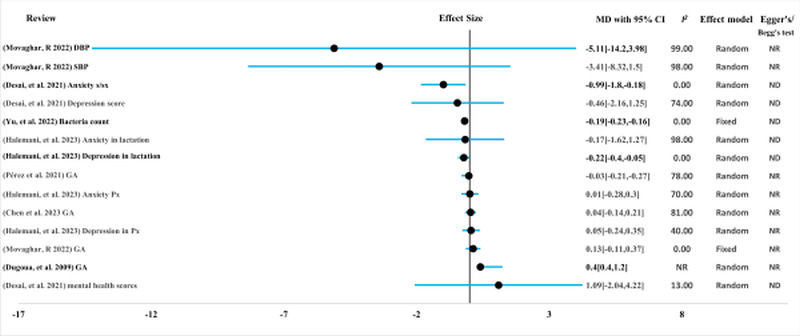
Effect of MBTIs on maternal clinical outcomes with continuous measurement (measured in mean difference (MD)). DBP – diastolic blood pressure, GA – gestational age, MBTI – microbiota-targeted interventions, ND – not done due to small studies, NR – not reported, Px – pregnancy, SBP – systolic blood pressure, S/Sx – sign and symptoms

C. Mastitis: The effect of microbiota-targeted interventions on lactational mastitis among breastfeeding women (n = 1197) was also evaluated and demonstrated a significant effect in lowering the incidence of mastitis, associated breast pain, and total bacterial count. (**Figure 5**, **Figure 6**). The review included in this theme was with moderate strength and certainty [48].

D. Maternal mental health: probiotics with multiple beneficial bacteria-species were administered to pregnant and lactating women and their impact on maternal mental health was evaluated in reviews (n = 2), which included 17 RCTs with 4020 participants [35,36] with moderate certainty. However, the majority of the findings showed no difference on maternal mental illness manifestations (**Figure 4**, **Figure 6**). Probiotics were useful for reducing anxiety symptoms during pregnancy and depression during lactation. However, evidence on the ability of prebiotics and synbiotics to support maternal mental health in the perinatal period is scarce, and further studies are highly recommended [35].

E. Hypertension control: pregnant women were supplemented with combinations of probiotic bacteria species and prebiotics (n = 214) in comparison with controls (n = 214) in five RCTs to measure the effectiveness of the combination of probiotic species in controlling hypertension during pregnancy. The certainty was moderate for this review [50]. The effects of MBTIs on diastolic and systolic blood pressure and the odds of preeclampsia were evaluated, and no difference was observed between the intervention and these outcomes were observed (**Figure 4**, **Figure 6**). This review by Movaghar et al. 2022 contains controversial findings on the mean differences and odds of the event occurring.

#### Effect of MBTIs on pregnancy outcome

Outcomes including gestational age at birth, preterm birth rate, caesarean section, low birth weight, macrosomia, small for gestational age, large for gestational age, miscarriage, and stillbirth were measured after MBTIs (mainly probiotic bacteria from the genera *Lactobacillus and Bifidobacterium*) in reviews of RCTs (n = 51) and a large number of participants (n = 8707). Although few of these outcomes (birth weight and gestational age) were significantly affected in one of the included reviews, the overall inference of these moderately certain reviews showed that MBTIs do not appear to influence perinatal outcomes [43,44].

#### Effect of MBTIs on infant outcomes

**Figure 7:**
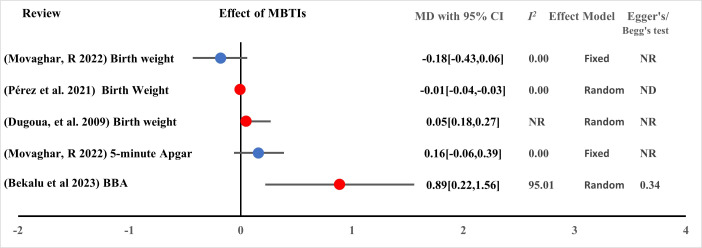
Effect of MBTIs on infant clinical outcomes with continuous measurement (measured in mean difference (MD)). BBA – beneficial bacteria abundance, MBTI – microbiota-targeted interventions, ND – not done due to small studies, NR – not reported.

A. Bacterial transfer to the infant: one of the targets of probiotic, prebiotic, and synbiotic administration during pregnancy and lactation is to achieve balanced bacterial transfer to the infant. In this section, we included all babies with no restriction to mode of birth and methodology of assessing infant gut microbiome. Babies’ mothers were supplemented with either probiotics in the intervention group or they were from the non-probiotics control groups. In view of addressing the offspring, three reviews were eligible and included in this umbrella review. Two of them had high levels of strength and certainty while the other was moderate. Although one review reported inconclusive findings, two recent reviews revealed that the infant gut microbiome is positively influenced by MBTIs (**Figure 7**) [51-53]. The administration of these interventions could increase the abundance of beneficial bacteria in infants’ guts.

**Figure 8:**
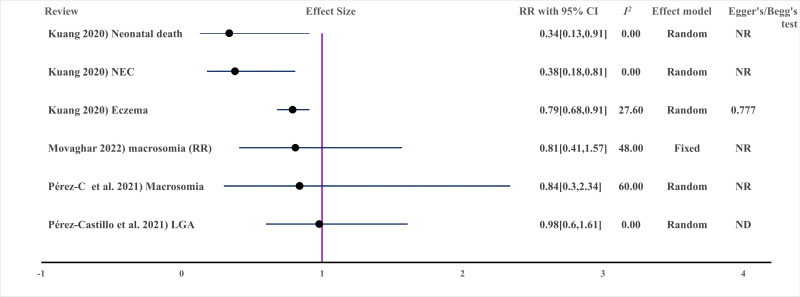
Effect of MBTIs on infant clinical outcomes with binary outcomes (measured in risk ratio (RR)). LGA – large for gestational age, ND – not done due to small studies, NR – not reported.

B. Infant allergy: two systematically screened and selected reviews with high and low certainty were included for measuring clinical outcomes such as atopic dermatitis, eczema, allergic disease, asthma, and sensitisation. Randomised controlled trials (n = 24) involving a total of 7619 pregnant and lactating women were included, and the results showed that probiotics administered to pregnant and lactating women were beneficial for treating atopic dermatitis, eczema, and related infant disorders [46,47]. MBTIs were also useful for reducing neonatal necrotising enterocolitis and death (**Figure 5**, **Figure 8**).

#### Safety of MBTIs

Interventions during pregnancy are curious and health professionals need to know the safety of the supplement. To this, an SRMA that had a moderate level of GRADE scale was incorporated in this overview of reviews that included RCTs (n = 8) with 1546 participants and reported that probiotics do not appear to pose any safety concerns, including an increasing incidence of pregnancy complications [49].

## DISCUSSION

This umbrella review summarised commonly used microbiota-targeted interventions during pregnancy and lactation, and their clinical implications and safety for mothers and babies. The commonly used microbiota-targeted interventions during pregnancy included probiotics followed by prebiotics. *Lactobacillus, Bifidobacterium, Streptococcus, Saccharomyces,* and *Anaerobes* were regularly used probiotics. This is supported by guidelines in which *Lactobacillus* and *Bifidobacterium* are the most common probiotic genera [26,60]. The commercially available products also incorporated the presence of two genera of beneficial bacteria, namely *Lactobacillus* and *Bifidobacterium*. Through the process of genotyping with a high throughput sequencing method such as metagenomic analysis revealed that *Lactobacillus* and *Bifidobacterium* bacteria constituted most of the composition within the products [61,62]. Similarly, in pregnancy, probiotics are common among other microbiota-targeted interventions with the main composition of these two dominant beneficial bacteria genera.

Microbiota-targeted interventions are very helpful for maternal glycaemic control, insulin metabolism, and balancing inflammatory and oxidative stress markers in gestational diabetes [31-34]. Although the exact mechanism of action of MBTIs on glycaemic control is unclear, it is articulated that low-grade chronic inflammation and decreased oxidative stress markers were associated with delayed evolution of glucose intolerance, hyperglycaemia, and hyperinsulinemia [63]. Probiotic bacteria strains such as *Lactobacillus GG* have antidiabetic effects by reducing the blood haemoglobin A1C and improving glucose tolerance [64]. Probiotics also modulate lipopolysaccharide-containing bacteria which can induce innate immunity in eukaryotes, thereby reducing inflammation and oxidative stress [65]. The effect of the intervention on reducing the risk of GDM was significant. The effect MBTIs measured in odds ratios also showed no difference on the odds of gestational diabetes mellitus than controls. Since RCTs are better measured in relative risk, taking their significant impact is worthwhile.

Moreover, MBTIs effectively reduce the incidence of rectovaginal group B *Streptococcus* colonisation [45], lactational mastitis [48], maternal anxiety symptoms [35,36] and infantile allergic disease [46,47]. Group B *Streptococcus* colonisation and mastitis prevention are associated with the whole-body microbial balance effect of interventions [27,66]. The importance of these interventions is beyond reducing the incidence of GBS, and reducing intrapartum antibiotic intake, which is one of the common causes of infant gut dysbiosis [67-69]. Microbiota-targeted interventions mainly the combination of probiotics and prebiotics also produce high levels of neurotransmitters, neuropeptides, and brain-derived neurotrophic factors, and improve central nervous system functions [70]. Nonetheless, no difference was observed in improving pregnancy outcomes and pregnancy-induced hypertension control [43,44].

The effect of MBTIs on infant outcomes such as bacterial transfer to offspring and the reduction of allergic diseases like eczema are very fundamental [51-53]. Since administered bacteria ameliorate breast milk, and translocate to the infant's gut [53], they reduce the hyperreactivity and inflammation of the infant skin and mucosal system by inhibiting allergens, interleukins and eosinophils, and tumour necrosis factors [71,72]. In general, perinatal MBTIs were effective at preventing infant allergies and remodelling the infant gut microbiome. This microbiome interplay is mediated mainly by breast milk, followed by exposure through the birth canal and transplacental transfer [73]. Due to microbiome exposure, immune imprinting (during pregnancy) and maturation periods (first 1000 days of life) occur in early life [74,75]. Early life microbiome maturation can be characterised by microbe acquisition, settlement, and selection with various functional features through time [75], and breastfeeding was the most important factor correlated with the microbiome structure [76]. The well-established early-life microbiome plays a pivotal role in the development of the host immune system, which coordinates host-microbe interactions. Disparities in microbiota-immunity interactions could contribute to the pathogenesis of immune-mediated disorders [77,78].

The safety of MBTIs was investigated by different studies, and it has been suggested that MBTIs are safe and helpful for various clinical outcomes among mothers and babies. No adverse reactions to probiotics have been reported thus far [49,79,80]. In the improvement of human health, MBTIs could be considered safe and cost-effective alternatives for the prevention of various diseases through colonisation, killing of pathogens, and immune induction to host cells [81-84].

This umbrella review has many strengths, as it is the first to summarise systematic reviews and meta-analyses of randomised controlled trials conducted on pregnant and lactating women aiming to assess the effects of probiotics, prebiotics, and synbiotics on maternal and infant clinical outcomes, pregnancy outcomes, and safety for mothers and babies. A large number of mother-infant pairs participated in the included trials. Since MBTIs are emerging platforms, this umbrella review presented a comprehensive conclusion as a steppingstone for clinical recommendations and researchers. The population, interventions, outcomes, and design of the studies were distinct or had no overlap; therefore, the findings presented could be plausible. The majority of included studies based on the GRADE system were categorised as moderate to high levels of certainty of evidence.

The limitations of this umbrella review are acknowledged, such as missing meta-data to summarise the common route of administration, influencing factors, prolonged health outcomes on babies, and composition of a variety of MBTIs. The other limitation of this study was the low methodological quality of the reviews included due to poor reporting. Almost all the reviews did not report on the funding sources of the RCTs they included. Again, the list of excluded studies and their brief reasons were missed in the majority of reviews included.

## CONCLUSIONS

**Figure 9:**
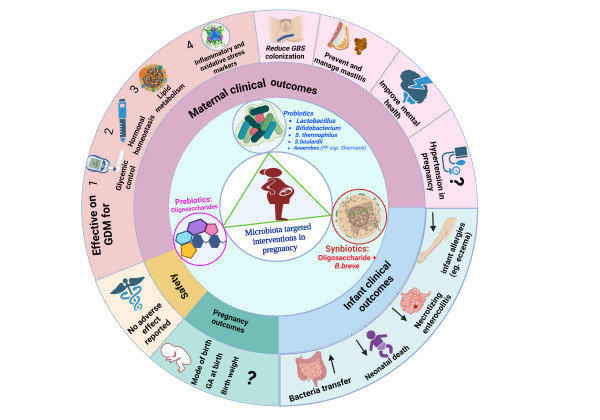
Common microbiota-targeted interventions (MTBIs) during pregnancy and their impact on different outcome categories (maternal and infant clinical outcomes, microbiome transfer, pregnancy outcomes, and safety) (? = controversial on its significant effect).

Our umbrella review revealed that the most commonly used MBTIsduring pregnancy and lactation were probiotics under the genera *Lactobacillus*, *Bifidobacterium, Streptococcus, Saccharomyces,* and *Anaerobes*. Thus far, probiotics, prebiotics, and synbiotics have been investigated and found to exhibit significant clinical importance in maternal glycaemic control; insulin metabolism; oxidative stress; inflammatory marker reduction; lactational mastitis treatment and prevention; anxiety symptom relief; and inhibition of group B* Streptococcus* colonisation. For infants, microbiota-targeted interventions were effective at remodelling the gut microbiome, preventing allergies including eczema and atopic dermatitis, preventing necrotising enterocolitis, and reducing neonatal mortality (**Figure 9**). However, reviews on the effect of microbiota-targeted interventions on controlling hypertension and influencing pregnancy outcomes such as preventing preterm birth showed no difference. The administration of perinatal maternal probiotics, prebiotics, or synbiotics was safe for both mothers and babies. The overall quality and certainty of the included studies were to the standard, the findings are considerably important. Further randomised clinical trials on the effect of MBTIs on maternal depressive symptoms, pregnancy outcomes, and prevention and management of preeclampsia are strongly recommended from this comprehensive umbrella review.

## Additional material


Online Supplementary Document

BT – bacterial transfer from mother to baby, BW – birth weight, CFU – colony forming unit, C/S – caesarean section, FBS – fasting blood glucose, GA – gestational age, GBS – group B *Streptococcus*, GDM – gestational diabetes mellitus, HTN – hypertension, LGA – large for gestational age, NEC – necrotizing enterocolitis, PB – preterm birth, SGA – small for gestational age
