# Supplementary material for: Microbiota-targeted interventions and clinical implications for maternal-offspring health: An umbrella review of systematic reviews and meta-analyses of randomised controlled trials
Source: J Glob Health. 2024 Sep 13;14:04177. doi: 10.7189/jogh.14.04177 (PMC11395958; doi:10.7189/jogh.14.04177)
Supplement: Online Supplementary Document [file jogh-14-04177-s001.pdf]

Supplementary Table 1. PRISMA 2020 Checklist

Section and Topic	Item #	Checklist item	Location where item is reported
**TITLE**			
Title	1	Identify the report as a systematic review.	1
**ABSTRACT**			
Abstract	2	See the PRISMA 2020 for Abstracts checklist.	2
**INTRODUCTION**			
Rationale	3	Describe the rationale for the review in the context of existing knowledge.	4-5
Objectives	4	Provide an explicit statement of the objective(s) or question(s) the review addresses.	5
**METHODS**			
Eligibility criteria	5	Specify the inclusion and exclusion criteria for the review and how studies were grouped for the syntheses.	6-7
Information sources	6	Specify all databases, registers, websites, organisations, reference lists and other sources searched or consulted to identify studies. Specify the date when each source was last searched or consulted.	6
Search strategy	7	Present the full search strategies for all databases, registers and websites, including any filters and limits used.	6
Selection process	8	Specify the methods used to decide whether a study met the inclusion criteria of the review, including how many reviewers screened each record and each report retrieved, whether they worked independently, and if applicable, details of automation tools used in the process.	6
Data collection process	9	Specify the methods used to collect data from reports, including how many reviewers collected data from each report, whether they worked independently, any processes for obtaining or confirming data from study investigators, and if applicable, details of automation tools used in the process.	7
Data items	10a	List and define all outcomes for which data were sought. Specify whether all results that were compatible with each outcome domain in each study were sought (e.g. for all measures, time points, analyses), and if not, the methods used to decide which results to collect.	7
	10b	List and define all other variables for which data were sought (e.g. participant and intervention characteristics, funding sources). Describe any assumptions made about any missing or unclear information.	--
Study risk of bias assessment	11	Specify the methods used to assess risk of bias in the included studies, including details of the tool(s) used, how many reviewers assessed each study and whether they worked independently, and if applicable, details of automation tools used in the process.	8
Effect measures	12	Specify for each outcome the effect measure(s) (e.g. risk ratio, mean difference) used in the synthesis or presentation of results.	9
Synthesis methods	13a	Describe the processes used to decide which studies were eligible for each synthesis (e.g. tabulating the study intervention characteristics and comparing against the planned groups for each synthesis (item #5)).	--
	13b	Describe any methods required to prepare the data for presentation or synthesis, such as handling of missing summary statistics, or data conversions.	--
	13c	Describe any methods used to tabulate or visually display results of individual studies and syntheses.	9

Section and Topic	Item #	Checklist item	Location where item is reported
	13d	Describe any methods used to synthesize results and provide a rationale for the choice(s). If meta-analysis was performed, describe the model(s), method(s) to identify the presence and extent of statistical heterogeneity, and software package(s) used.	9
	13e	Describe any methods used to explore possible causes of heterogeneity among study results (e.g. subgroup analysis, meta-regression).	NA
	13f	Describe any sensitivity analyses conducted to assess robustness of the synthesized results.	NA
Reporting bias assessment	14	Describe any methods used to assess risk of bias due to missing results in a synthesis (arising from reporting biases).	NA
Certainty assessment	15	Describe any methods used to assess certainty (or confidence) in the body of evidence for an outcome.	8
**RESULTS**			
Study selection	16a	Describe the results of the search and selection process, from the number of records identified in the search to the number of studies included in the review, ideally using a flow diagram.	10
	16b	Cite studies that might appear to meet the inclusion criteria, but which were excluded, and explain why they were excluded.	--
Study characteristics	17	Cite each included study and present its characteristics.	12-15
Risk of bias in studies	18	Present assessments of risk of bias for each included study.	10
Results of individual studies	19	For all outcomes, present, for each study: (a) summary statistics for each group (where appropriate) and (b) an effect estimate and its precision (e.g. confidence/credible interval), ideally using structured tables or plots.	10-12
Results of syntheses	20a	For each synthesis, briefly summarise the characteristics and risk of bias among contributing studies.	10
	20b	Present results of all statistical syntheses conducted. If meta-analysis was done, present for each the summary estimate and its precision (e.g. confidence/credible interval) and measures of statistical heterogeneity. If comparing groups, describe the direction of the effect.	NA
	20c	Present results of all investigations of possible causes of heterogeneity among study results.	NA
	20d	Present results of all sensitivity analyses conducted to assess the robustness of the synthesized results.	NA
Reporting biases	21	Present assessments of risk of bias due to missing results (arising from reporting biases) for each synthesis assessed.	NA
Certainty of evidence	22	Present assessments of certainty (or confidence) in the body of evidence for each outcome assessed.	10
**DISCUSSION**			
Discussion	23a	Provide a general interpretation of the results in the context of other evidence.	16-18
	23b	Discuss any limitations of the evidence included in the review.	18-19
	23c	Discuss any limitations of the review processes used.	19
	23d	Discuss implications of the results for practice, policy, and future research.	19

Section and Topic	Item #	Checklist item	Location where item is reported
**OTHER INFORMATION**			
Registration and protocol	24a	Provide registration information for the review, including register name and registration number, or state that the review was not registered.	2&6
	24b	Indicate where the review protocol can be accessed, or state that a protocol was not prepared.	6
	24c	Describe and explain any amendments to information provided at registration or in the protocol.	NA
Support	25	Describe sources of financial or non-financial support for the review, and the role of the funders or sponsors in the review.	20
Competing interests	26	Declare any competing interests of review authors.	20
Availability of data, code and other materials	27	Report which of the following are publicly available and where they can be found: template data collection forms; data extracted from included studies; data used for all analyses; analytic code; any other materials used in the review.	20

NA: not applicable.

From: Page MJ, McKenzie JE, Bossuyt PM, Boutron I, Hoffmann TC, Mulrow CD, et al. The PRISMA 2020 statement: an updated guideline for reporting systematic reviews. BMJ 2021;372:n71. doi: 10.1136/bmj.n71

Supplementary materials

Supplementary Table 2: Searching terms and outputs for six databases.

**Database**	**Terms**	**Result**
PubMed	((Probiotic* OR Prebiotic* OR Synbiotic* OR ("Probiotic* OR Prebiotic* OR Symbiotic*" [MeSH] OR (“Probiotic* OR Prebiotic* OR Synbiotic*” [All Fields]))) AND (Pregnancy OR Lactation OR Antepartum OR Prenatal OR Perinatal OR Postpartum OR (“Pregnancy”[MeSH] OR (“Pregnancy” [All Fields]))) AND (Preeclampsia OR Hypertension disorders OR Gestational diabetes mellitus OR Premature rupture of membrane OR Mastitis OR Breast engorgement OR Mental disorder OR Psychosis OR Depression OR Anxiety OR Safety OR Pregnancy outcome OR Neonatal outcome OR Infection OR Preterm-labor OR Abortion OR Miscarriage OR (“Complication* OR Outcome*” [All Fields])) AND (Systematic review and meta-analysis OR Review OR meta-analysis))	456
Web of Science	((Probiotic* OR Prebiotic* OR Synbiotic*) AND (Pregnancy OR Lactation OR Antepartum OR Prenatal OR Perinatal OR Postpartum) AND (Preeclampsia OR Hypertension disorders OR Gestational diabetes mellitus OR Premature rupture of membrane OR Mastitis OR Breast engorgement OR Mental disorder OR Psychosis OR Depression OR Anxiety OR Safety OR Pregnancy outcome OR Neonatal outcome OR Infection OR Preterm-labor OR Abortion OR Miscarriage) AND (Systematic review and meta-analysis OR Review OR meta-analysis))	380
Cochrane Library	(((Probiotics) OR (Prebiotics)OR (synbiotics) OR (symbiotics)) AND ((Systematic review and meta-analysis) OR ("meta-analysis") OR (review)) AND ((Pregnancy) OR (lactation) OR (pregnancy outcome) OR (infant outcome))) in Title Abstract Keyword - (Word variations have been searched)	24
EMBASE	(((Probiotics) OR (Prebiotics)OR (synbiotics) OR (symbiotics)) AND ((Systematic review and meta-analysis) OR ("meta-analysis") OR (review)) AND ((Pregnancy) OR (lactation) OR (pregnancy outcome) OR (infant outcome)))	554
Science Direct	((Probiotics OR Prebiotics OR Synbiotics) AND (Pregnancy OR Lactation) AND (Systematic review and meta-analysis OR Review OR meta-analysis))	928
Scopus	((probiotic* OR prebiotic* OR synbiotic*) AND (pregnancy OR lactation) AND (systematic AND review AND meta-analysis OR review OR meta-analysis))	240
Total		2582

Supplementary Table 3: Certainty of evidence included in the umbrella review using the GRADE system.

Review	Risk of bias	Inconsistency	Indirectness	Imprecision	Publication bias	Certainty
Othman, et al. 2007	Serious	Not applicable	No serious	No serious	Not reported	●●●○
Dugoua, et al. 2009	Not reported	No serious	No serious	No serious	Unlikely	●●●○
Han, et al. 2019	No serious	No serious	No serious	No serious	Unlikely	●●●●
Masulli et al. 2020	No serious	No serious	No serious	No serious	Likely	●●●○
Kuang, L., Y. 2020	No serious	No serious	No serious	No serious	Unlikely	●●●●
Moore, et al. 2020	No serious	Not applicable	No serious	No serious	Not applicable	●●●●
Desai, et al. 2021	No serious	No serious	No serious	No serious	Not done	●●●○
Pérez et al. 202	Serious	No serious	No serious	No serious	Unlikely	●●●○
Chen, et al. 2022	No serious	Serious	No serious	No serious	Likely	●○○○
Menichini, D. 2022	Serious	No serious	No serious	No serious	Unlikely	●●●○
Colquitt, et al. 2022	No serious	Not reported	No serious	Not reported	Likely	●○○○
Yu, et al. 2022	No serious	No serious	No serious	No serious	Not done	●●●○
Mahdizade et al. 2022	No serious	No serious	No serious	No serious	Likely	●●●○
Movaghar, R 2022	No serious	Serious	No serious	No serious	Not reported	●●●○
Martin, et al. 2022	No serious	Not applicable	No serious	No serious	Not applicable	●●●●
Halemani, et al. 2023	No serious	Serious	No serious	No serious	Not reported	●●●○
Bekalu et al 2023	No serious	Serious	No serious	No serious	Unlikely	●●●○

SRMA: Systematic Review and Meta-analysis; SR: Systematic Review; GDM: Gestational diabetes Mellitus; SBP: Systolic blood pressure; DBP: Diastolic blood pressure; MA: Meta-analysis; RoB: Risk of bias; NR: Not reported; ●●●● high; ●●●○ Moderate; ●○○○ Low.

Supplementary Table 4: Graphical crosstabulation to display the overlapping and selection of studies included in Gestational diabetes theme. Blue highlighted reviews are selected after overlapping evaluation while green boxes indicate original articles in the respective reviews.

	(Zheng, Feng et al. 2018)	(Pan, Pan et al. 2019)	(Han, Sun et al. 2019)	(Masulli, et al. 2020)	(Lagowska, et al. 2020)	(Okesene-Gafa, et al. 2020)	(Hasain, Che et al. 2021)	(Ramanathan, et al. 2021)	(Pan, Zheng et al. 2021)	(Zhou, Ding et al. 2021)	(Davidson, et al. 2021)	(Cetinkaya, et al. 2022)	(Mahdizade Ari, et al. 2022)	(Obuchowska, et al. 2022)	(Pakmehr, et al. 2022)	(Chen et al. 2022)	(Chu, Yan et al. 2022)	(Kaminska, et al. 2022)	(Yefet, Bar et al. 2023)	(Mu, Guo et al. 2023)
Laitinen et al. 2009																				
Allen, 2010																				
Luoto et al. 2010																				
Brantsæter et al. 2011																				
Ilmonen et al 2011																				
Asemi 2012																				
Raakel Luoto, 2012																				
Asemi et al 2013																				
Marloes Nitert1 2013																				
Hoppu 2014																				
Lindsay et al. 2014																				
Taghizadeh etal 2014																				
afarnejad KL 2015																				
Dolatkhah 2015																				
Lindsay 2015																				
Ahmadi et al. 2016																				
Barthow 2016																				
Jafarnejad 2016																				
Jamilian et al. 2016																				
Karamali 2016																				
Luisa F, 2016																				
Sofie Ingdam Halkjaer, 2016																				
Kristin 2017																				
Luisa F, 2017																				

Shaun Sabico, 2017																				
Wickens et al. 2017																				
Badehnoosh 2018																				
Hajifaraji 2 2018																				
Karamali, 2018																				
Nabhani et al. 2018																				
Nordqvist et. al. 2018																				
Asgharian 2019																				
Babadi 2019																				
Callaway 2019																				
Ebrahim etr. al, 2019																				
Jamilian 2019																				
Kijmanawat 2019																				
Lihui Si, 2019																				
Okesene- et al., 2019																				
Oksene-Gafa 2019																				
Pellonperä, 2019																				
Sahhaf 2019																				
Asgharian 2020																				
Halkjaer et al 2020																				
Godfrey et al 2021																				
Reyes- et al. (2021)																				
Shahriari et al 2021																				
Amirani et al., 2022																				
Baloch 2022																				

Supplementary Table 5: Graphical crosstabulation to display the overlapping and selection of studies included in Group B Streptococcus theme.

	Jois, R. S. 2020	Menichini, D. 2022
Hanson et al 2014		
Ho et al 2016		
Ming-Ho, 2016		
Olsen et al 2018		
Azizet al 2018		
Sharpe et al., 2019		
Farr, 2020		

Supplementary Table 6: Graphical crosstabulation to display the overlapping and selection of studies included in the preeclampsia theme.

	Movaghar, R 2022
Kopp 2008	
Yuniati, 2013	
García-Ródenas, 2016	
Cabana 2017	
Hajifaraji 2 2018	

Supplementary Table 7: Graphical crosstabulation to display the overlapping and selection of studies included in the infant allergies theme.

	(Colquitt, et al. 2022)	(Yin, He et al. 2019)	(Kuang 2020)	(Doege, et al. 2012)	(Voigt 2022)	(Amalia, et al. 2020)
Kalliomäki et al 2001			■		■	
Rautava et al. 2002					■	
Kalliomäki et al. 2003					■	
Kukkonen et al. 2006			■			
Abrahamsson et al. 2007			■	■		
Kukkonen et al 2007			■	■	■	
Kallioma¨kietal. 2007				■	■	
Huurre et al. 2008	■	■			■	
Bottcher 2008		■				
Kopp 2008		■	■	■	■	■
Prescott 2008		■				
Wickens 2008		■		■	■	■
Samanta et al 2008			■			
Hurreeetal. 2008				■		
Niers 2009		■	■			■
Soh 2009		■				■
Kuitunen 2009		■		■	■	■
West 2009		■				■
Kim 2009						■
Boyle et al. 2010	■		■			■
Dotterud et al. 2010	■	■	■		■	■
Kim 2010		■	■			
Wu Fuling, 2010		■				
Allen et al.2010			■			
Luoto et al. 2010			■			
Boyle 2011		■				
Ou 2012		■	■		■	■
Rautava 2012		■			■	■
Wickens 2012		■	■		■	■
Jensen 2012		■				
Abrahamsson 2013		■				
Wickens 2013		■			■	■
West 2013		■				■
Benor et al. 2013			■			
Allen 2014		■				■
Lindsay et al 2014			■			
Nomoto,et al.2014					■	
Bertelsen,et al. 2014						■
Simpson et al. 2015	■				■	■
Mastromarino et al. 2015			■			
Cabana 2017		■				
Wickens et al. 2017			■			
Peldan et al. 2017					■	
amm,et al.2017						■
Wickens et al. 2018	■				■	
Davies,et al.2018						■
Rautava et al. 2021	■					

Supplementary Table 8: Graphical crosstabulation to display the overlapping and selection of studies included in the lactation mastitis theme.

<table>
<tr><th></th><th>(Barker, et al. 2020)</th><th bgcolor="#00B0F0">(Yu, et al. 2022)</th></tr>
<tr><td>Jime´nez et al. 2008</td><td bgcolor="#A9D08E"></td><td bgcolor="#A9D08E"></td></tr>
<tr><td>Arroyo et al. 2010</td><td bgcolor="#A9D08E"></td><td bgcolor="#A9D08E"></td></tr>
<tr><td>MaldonadoLobo´n et al. 2015</td><td bgcolor="#A9D08E"></td><td bgcolor="#A9D08E"></td></tr>
<tr><td>Ferna´ndez et al. 2016</td><td bgcolor="#A9D08E"></td><td bgcolor="#A9D08E"></td></tr>
<tr><td>Hurtado et al. 2017</td><td bgcolor="#A9D08E"></td><td bgcolor="#A9D08E"></td></tr>
<tr><td>Jime´nez et al. 2021</td><td></td><td bgcolor="#A9D08E"></td></tr>
</table>

Supplementary Table 9: Graphical crosstabulation to display the overlapping and selection of studies included in the mental health theme.

<table>
<tr><th></th><th bgcolor="#00B0F0">(Desai, et al. 2021)</th><th>(Trifkovič, et al. 2022)</th><th bgcolor="#00B0F0">(Halemani, et al. 2023)</th></tr>
<tr><td>Mi, 2015</td><td></td><td bgcolor="#A9D08E"></td><td></td></tr>
<tr><td>Mastromarino, 2015</td><td></td><td></td><td bgcolor="#A9D08E"></td></tr>
<tr><td>Mirghafourvand et al 2016</td><td bgcolor="#A9D08E"></td><td bgcolor="#A9D08E"></td><td></td></tr>
<tr><td>Slykerman et al 2016</td><td bgcolor="#A9D08E"></td><td></td><td></td></tr>
<tr><td>Slykerman, 2017</td><td></td><td bgcolor="#A9D08E"></td><td bgcolor="#A9D08E"></td></tr>
<tr><td>Korpela, 2018</td><td></td><td></td><td bgcolor="#A9D08E"></td></tr>
<tr><td>Okesene-Gafa et al. 2019</td><td bgcolor="#A9D08E"></td><td></td><td></td></tr>
<tr><td>Dawe et al. 2020</td><td bgcolor="#A9D08E"></td><td bgcolor="#A9D08E"></td><td bgcolor="#A9D08E"></td></tr>
<tr><td>Hulkkonen, 2021</td><td></td><td bgcolor="#A9D08E"></td><td></td></tr>
<tr><td>Browne, 2021</td><td></td><td bgcolor="#A9D08E"></td><td bgcolor="#A9D08E"></td></tr>
<tr><td>Hulkkonen, 2022</td><td></td><td></td><td bgcolor="#A9D08E"></td></tr>
<tr><td>Yıldız Karaahme, 2022</td><td></td><td></td><td bgcolor="#A9D08E"></td></tr>
</table>

Supplementary Table 10: Graphical crosstabulation to display the overlapping and selection of studies included in the pregnancy outcome theme.

	(Othman, et al. 2007)	(Dugoua, et al. 2008)	(Grev, et al. 2017)	(Jarde, et al. 2018)	(Pérez et al. 2021)	Bekalu et al 2023
Neri 1993	■					
Kalliomaki 2001		■		■	■	
Rautava etal. 2002		■				
Nishijima 2005	■	■				
Gueimonde etal. 2006		■				
Kukkonen etal. 2006		■				
Abrahamsson 2007		■		■	■	
Kaplas etal. 2007		■				
Kukkonen etal. 2007		■			■	
Kopp 2008			■		■	
Bergmann, 2008				■		
Bababi et al 2008					■	
Wickens et al 2008					■	
Laitinen 2009			■	■	■	
Niers, 2009				■	■	
Kim, 2010				■	■	
Allen, 2010				■	■	
Dotterund 2010				■	■	
Luoto 2010	■					■
Asemi 2011				■		
Boyle 2011				■	■	
Krauss-Silva 2011				■		
Ou 2012			■	■	■	
Rautava 2012			■	■		
Hantoushzadeh, 2012				■		
Taghizadeh et al 2013					■	
Lindsay 2014			■	■	■	
Benor 2014			■			
Lindsay 2015			■	■		
Dolatkhah 2015			■	■	■	
Mastromarino 2015				■	■	■
Okesene-Gafa 2016						
Fernández 2016			■		■	
Jacobsson 2016			■			
Mantaring 2016			■			
Karamali, 2016				■	■	
Jafarnejad 2016				■	■	
Ho 2016				■	■	
Ahmadi et al 2016					■	
Gille et al 2016					■	

Jamilian et al 2016						
Baldassarre 2016						
Dewanto et al 2017						
Wickens et al. 2017						
Vähämiko et al 2017						
Karamali 2018						
Badehnoosh 2018						
Mantaring et al 2018						
McMillan et al 2018						
Nabhani et al. 2018						
Okense-Gafa et al 2018						
Olsen et al. 2018						
Kijmanawa 2019						
Callaway 2019						
Asgharian et al.2019						
Chen et al 2019						
Jamilian et al 2019						
Pellonperä et. 2019						
Sahhaf et al 2019						
Sharpe et al. 2019						
Ali Pourmirzaiee et al 2020						
Halkjær et al. 2020						
Yang et al. 2020						
Pastor et al., 2020						
Karaahmet 2022						

Supplementary Table 11: Graphical crosstabulation to display a study included in the safety theme with no overlap.

	(Dugoua, et al. 2009)
Kalliomäki et al. 2001	
Rautava et al. 2002	
Nishijima et al. 2005	
Gueimonde et al.2006	
Kukkonen et al. 2006	
Abrahams son et al. 2007	
Kaplas et al. 2007	
Kukkonen et al. 2007	

Supplementary Table 12: Graphical crosstabulation to display the overlapping and selection of studies included in the bacterial transfer to infant theme.

	(Moore, et al. 2020)	(Martin et al. 2022)	bekalu et al 2023
Lahtinen et al 2009	■		
Abrahamsson et al, 2009			■
Grzeskowiak L et al 2012			■
Yuniati, 2013		■	
Enomoto T 2014			■
Dotterud et al 2015	■		■
Rutten et al 2015	■		
Mastromarino, 2015		■	■
Baglatzi, 2016		■	
Cooper, 2016		■	
García-Ródenas, 2016		■	
Bazanella, 2017		■	
Chien Chua, 2017		■	
Frese, 2017		■	
Korpela, 2018		■	
Hurkala, 2020		■	
Pastor-V et al, 2020			■
Estorninos, 2021		■	
Phavichitr, 2021		■	

	(Hasain, Che Roos et al. 2021)	Cetinkaya et al. 2022)	Mahdizade et al. 2022)	(Masulli, Vitacolonna et al. 2020)	(Obuchowska, Gorczyca et al. 2022)	(Yefet, Bar et al. 2023)	(Zheng, Feng et al. 2018)	(Mu, Guo et al. 2023)	(Pan, Pan et al. 2019)	(Ramanathan, Jagadeesh et al. 2021)	(Lagowska, Malinowska et al. 2020)	(Pakmehr, Ejtahed et al. 2022)	(Zhang, Niu et al. 2023)	(Pan, Zheng et al. 2021)	(Okesene-Gafa, Moore et al. 2020)	(Zhou, Ding et al. 2021)	(Davidson, Barrett et al. 2021)	(Chu, Yan et al. 2022)	(Han, Sun et al. 2019)	(Kaminska, Stenclik et al. 2022)
Cetinkaya et al. 2022	50.00%																			
(Mahdizade Ari, Teymouri [illegible]	35.70%	28.60%																		
(Masulli, Vitacolonna et al. 2020)	0.00%	0.00%	10.25%																	
(Obuchowska, Gorczyca et al. 2022)	25.00%	38.50%	17.60%	25.00%																
(Yefet, Bar et al. 2023)	60.00%	57.00%	53.50%	5.00%	33.00%															
(Zheng, Feng et al. 2018)	25.00%	11.00%	18.75%	0.00%	0.00%	20.00%														
(Mu, Guo et al. 2023)	75.00%	72.72%	39.29%	0.00%	31.25%	78.57%	23.53%													
(Pan, Pan et al. 2019)	33.33%	27.27%	17.24%	0.00%	6.67%	25.00%	23.10%	30.76%												
(Ramanathan, Jagadeesh et al. 2021)	35.71%	21.43%	23.33%	0.00%	5.56%	27.78%	46.15%	33.33%	50.00%											
(Lagowska, Malinowska et al. 2020)	47.06%	43.75%	48.28%	11.11%	19.10%	52.63%	38.89%	62.50%	23.53%	41.18%										
(Pakmehr, Ejtahed et al. 2022)	0.00%	0.00%	15.15%	25.00%	11.11%	4.35%	11.11%	0.00%	6.67%	11.76%	4.17%									
(Zhang, Niu et al. 2023)	0.00%	0.00%	9.68%	22.22%	14.29%	5.26%	0.00%	0.00%	9.10%	7.14%	0.00%	33.33%								
(Pan, Zheng et al. 2021)	36.36%	33.33%	50.00%	19.10%	25.00%	84.62%	36.36%	47.62%	18.18%	31.82%	66.67%	20.00%	13.04%							
(Okesene-Gafa, Moore et al. 2020)	46.15%	41.67%	32.14%	0.00%	18.75%	43.75%	18.75%	42.86%	36.36%	35.71%	33.33%	5.56%	7.14%	26.10%						
(Zhou, Ding et al. 2021)	66.67%	80.00%	37.90%	0.00%	29.41%	62.50%	22.22%	64.28%	20.00%	31.25%	50.00%	0.00%	0.00%	39.13%	61.54%					
(Davidson, Barrett et al. 2021)	0.00%	0.00%	12.90%	50.00%	21.43%	5.00%	21.43%	0.00%	8.30%	13.33%	10.00%	23.53%	30.00%	28.57%	6.67%	5.56%				
(Chu, Yan et al. 2022)	0.00%	0.00%	13.79%	50.00%	25.00%	5.56%	7.14%	0.00%	0.00%	0.00%	11.11%	36.36%	22.22%	19.10%	0.00%	0.00%	9.10%			
(Han, Sun et al. 2019)	27.78%	16.67%	20.59%	5.88%	4.55%	22.73%	64.29%	26.32%	26.67%	46.67%	40.00%	9.53%	0.00%	32.00%	22.22%	25.00%	17.65%	5.88%		
(Kaminska, Stenclik et al. 2022)	6.25%	0.00%	12.90%	20.00%	13.33%	10.53%	17.65%	5.88%	8.33%	14.29%	4.76%	30.77%	30.00%	22.73%	14.29%	5.56%	40.00%	20.00%	17.65%	

0-5%	slight overlap
6-10%	Moderate overlap
	High overlap
	Very high overlap

Supplementary Figure 1: Citation matrix for overlapping management in GDM.
